# Hémothorax spontané: complication rare de la neurofibromatose type 1

**DOI:** 10.11604/pamj.2017.28.85.13820

**Published:** 2017-09-27

**Authors:** Soumia Fdil, Saad Bouchikhi, Jamal-Eddine Bourkadi

**Affiliations:** 1 Service de Pneumologie, Hopital Moulay Youssef, Faculté de Médecine et de Pharmacie, Université Mohammed V, CHU Ibn Sina, 10000 Rabat, Maro

**Keywords:** Hémothorax, spontané, la neurofibromatose de type 1, Hemothorax, spontaneous, neurofibromatosis type 1

## Abstract

La neurofibromatose de type 1 (NF1) ou maladie de Von Recklinghausen est une maladie héréditaire la plus fréquente des phacomatoses, de transmission autosomique dominante. Ses complications pulmonaires sont rarement décrites dans la littérature. Les complications vasculaires sont retrouvées chez 3,6% des patients. Nous rapportons le cas d'une patiente âgée de 38 ans, suivie pour neurofibromatose type 1, admise aux urgences dans un tableau de choc hémorragique, avec à l'examen plusieurs taches café au lait, de nombreux neurofibromes plexiformes, un syndrome d'épanchement liquidien gauche. La ponction pleurale a objectivé un liquide hémorragique coagulable, la patiente a bénéficié d'une transfusion et de drainage thoracique en urgence. Le bilan était complété par un angioscanner qui n'a pas objectivé d'embolie pulmonaire ni d'autres lésions associées. L'hémothorax spontané est une complication rare et grave de la neurofibromatose, il est probablement du à l'atteinte vasculaire de cette maladie.

## Abstract (en)

Neurofibromatosis type 1 (NF1), also known as Von Recklinghausen’s disease is an autosomal dominant genetic disorder. It is the most common of phacomatoses. Pulmonary complications have been rarely described in the literature. Vascular complications have been reported in 3.6% of patients. We here report the case of a 38-year old female patient, followed-up for neurofibromatosis type 1, admitted to the Emergency Department with hemorrhagic shock. Clinical examination showed several coffee-with-milk colored spots, many plexiform neurofibromas, left-sided pleural effusion syndrome. Pleural puncture objectified coagulable haemorrhagic fluid. The patient received transfusion and emergency chest drainage. Patient’s assessment was completed by angioscanner which showed no pulmonary embolism or other associated lesions. Spontaneous hemothorax is a rare and severe complication of neurofibromatosis. It is probably due to vascular injury caused by this disease.

## Introduction

La neurofibromatose type 1 (NF1) ou maladie de Von Recklinghausen est une pathologie à déterminisme génétique autosomique dominant dont la pénétrance est quasi complète avant l'âge de 5 ans. Son incidence est de 1/3000 naissances [1]. L'expression est très variable au sein d'une même famille et implique un suivi régulier, adapté à l'âge à fin de prendre en charge les complications .Cette maladie est associée à une atteinte multi-organique, principalement le système nerveux central. Cependant, l'atteinte vasculaire, comme l'occlusion, la sténose, l'anévrisme ou l'ectasie, est rare [2]. Nous rapportons un cas d'hémothorax spontané chez une patiente de 38 ans connue porteuse d'une NF1.

## Patient et observation

**Figure 1:**
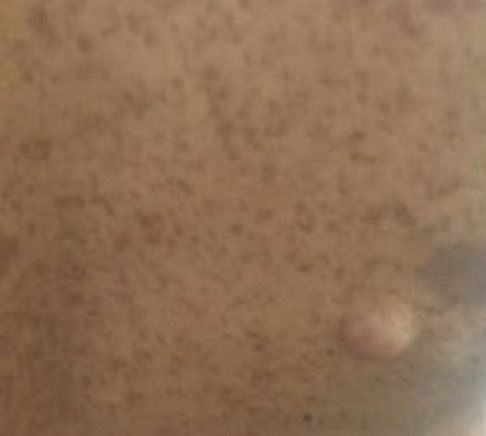
Taches café-au-lait et neurofibrome dermique du dos

**Figure 2:**
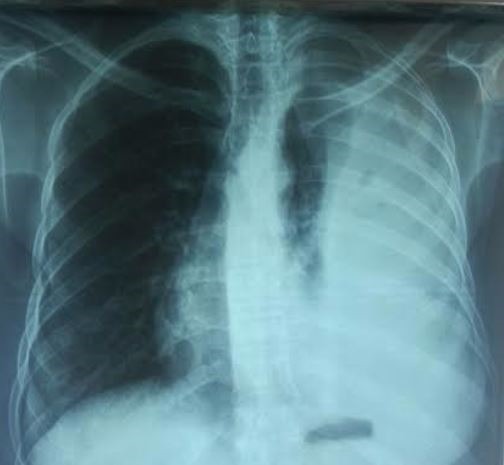
Radiographie thoracique de face: épanchement pleural gauche de grande abundance

**Figure 3:**
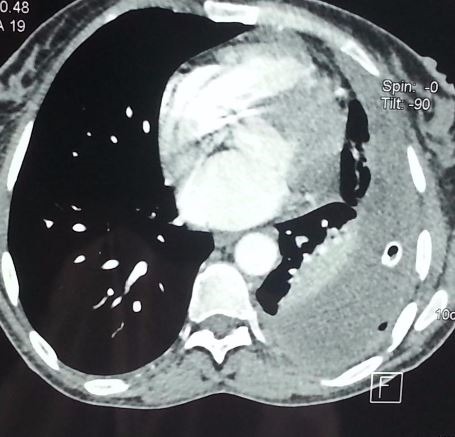
TDM thoracique coupe axiale médiastinale: hémothorax gauche drainé

**Figure 4:**
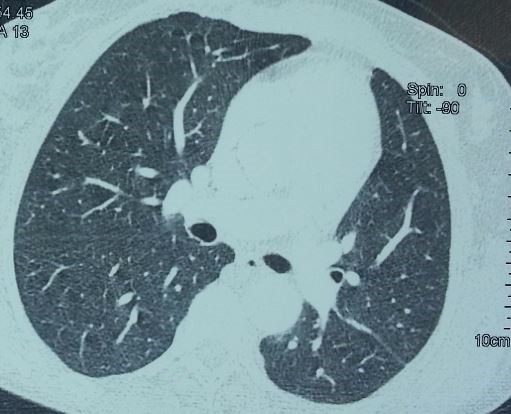
TDM thoracique coupe axiale parenchymateuse: parenchyme normal après décortication

Il s'agit d'une patiente âgée de 38 ans, suivie pour NF1 (Figure 1) avec notion de cas similaires dans la famille, connue hypertendue il y a 2 ans sur polykystose rénale bilatérale , cette patiente était admise aux urgences dans un tableau de choc hémorragique, l'examen clinique a objectivé une hypotension à 70/40 mmHg, une pâleur cutanéo-muqueuse, plusieurs taches café-au-lait et neurofibromes plexiformes sur le tronc, les membres et le visage, une polypnée à 28 c/min et un syndrome d'épanchement liquidien gauche. Le bilan biologique trouve une anémie à 3,4 g/dl. La radiographie thoracique (Figure 2) a confirmé la présence d'une pleurésie, dont la ponction a montré un liquide hématique coagulable. Une transfusion en urgence de trois culots globulaires était réalisée avec drainage thoracique. Un angioscanner thoracique a permis d'éliminer l'embolie pulmonaire (Figure 3), et a montré uniquement l'épanchement liquidien pleural sans autres lésions associées avec reins hypertrophiés multikystiques. L'évolution était marquée par la surinfection de l'hémothorax et la patiente a bénéficié d'une décortication avec bonne évolution (Figure 4).

## Discussion

La neurofibromatose de type 1 est une maladie héréditaire la plus fréquente des phacomatoses de transmission autosomique dominante [2]. Le gène de NF1 dont les mutations prédisposent à la maladie est localisé sur le bras long du chromosome 17 [3] et aboutissent à une activation non contrôlée de la voie des MAP kinase avec dérégulation des mécanismes de prolifération et différenciation cellulaire. Cette activation de la voie des MAP kinases pourrait contribuer au développement de tumeurs bénignes ou malignes des gaines nerveuses, des hypertensions pulmonaires par hyperplasie des cellules musculaires lisses vasculaires des artères pulmonaires et des atteintes interstitielles (cellules fibroblastiques) [4]. Les manifestations vasculaires de la NF1 ont été décrites pour la première fois par Reubi [5] en 1944 elles peuvent être fatales et sont retrouvées chez 3,6% des patients NF1. Norton et al. [6] ont démontré par immunohistochimie l'expression de la neurofibromine dans la paroi des vaisseaux cérébraux, rénaux et dans l'aorte de rats et de bœufs, et ont conclu qu'il est possible que la réduction de l'expression de la neurofibromine à ce niveau soit responsable de l'incidence élevée de l'atteinte vasculaire chez les patients NF1. En outre, l'étude histologique de parois de vaisseaux altérés chez des patients NF1 avait montré une prolifération intimale de cellules fusiformes avec fibrose, formation d'anévrismes et réduction de la couche élastique. A cela s'ajoute les occlusions de petites artères, les thromboses, les ectasies, ou les malformations cardiaques. L'hémothorax est une complication qui peut être fatale notamment lorsque les malformations touchent les gros vx [7]. Les artères sous-claviculaires et intercostales sont les plus sujettes au saignement. Leier et al. [4] ont décrit deux causes de rupture artérielle. Tout d'abord, la neurofibromatose envahit la couche musculaire, ce qui peut réduire le renforcement de la paroi vasculaire, deuxièmement, le vasa vasorum d'un grand vaisseau peut être comprimé par un tissu neurofibromateux, ce qui entraîne un segment fragile de l'artère secondaire à l'ischémie. Le traitement endovasculaire à type d'embolisation ou de stent reste le traitement de choix en cas de saignement important, un complément par un traitement chirurgical à visée hémostatique peut être nécessaire [3]. Un cas d'hémothorax massif gauche sur rupture d'anévrysme de l'artère intercostale proximale gauche était rapporté par Hongsakul en 2013, ayant bénéficié d'une embolisation en urgence [6]. En ce qui concerne notre observation l'hypothèse la plus probable est la rupture de petits vaisseaux fragiles au niveau de la cavité pleurale chez notre patiente porteuse de Nf1 qui a bien évolué sous drainage seul. Récemment l'hypertension artérielle pulmonaire pré-capillaire s'est révélée être une complication rare et sévère, jusqu'à 60 % des adultes ayant une HTP [8,9] d'où la nécessité de faire une ETT au moins une fois par an. Devant l'absence de certitude concernant l'étiologie de cette hypertension pulmonaire la neurofibromatose de type 1 est listée dans le groupe 5.

## Conclusion

Notre observation illustre un cas d'hémothorax spontané, complication entrant dans le cadre de vasculopathie due à la neurofibromatose type 1, et permet d'attirer l'attention sur le risque hémorragique élevé chez les neurofibromateux et de suggérer l'existence d'une relation non fortuite entre le développement d'hémothorax spontané et la NF1.

## Conflits d’intérêts

Les auteurs ne déclarent aucun conflit d'intérêts.
